# Learning best-practices in journalology: course description and attendee insights into the inaugural EQUATOR Canada Publication School

**DOI:** 10.1186/s12919-018-0155-4

**Published:** 2018-08-23

**Authors:** Jacqueline Galica, Alyssandra Chee-a-tow, Shikha Gupta, Atul Jaiswal, Andrea Monsour, Andrea C. Tricco, Kelly D. Cobey, Nancy J. Butcher

**Affiliations:** 1 School of Nursing, Faculty of Health Sciences, Queen’s University, Kingston, Canada; 2 Child Health Evaluative Sciences, The Hospital for Sick Children Research Institute, Toronto, Canada; 3 School of Rehabilitation Therapy, Queen’s University, Kingston, Canada; 4 Epidemiology Division, Dalla Lana School of Public Health, University of Toronto, Toronto, Canada; 5 Li Ka Shing Knowledge Institute of St. Michael’s Hospital, Toronto, Canada; 6 Centre for Journalology, Ottawa Hospital Research Institute, Ottawa, Canada; 7 School of Epidemiology and Public Health, University of Ottawa, Ottawa, Canada; 8 Department of Psychology, University of Stirling, Stirling, Scotland

**Keywords:** Medical education, Publication science, Scholarly communications, Reporting quality

## Abstract

**Background and purpose:**

Dissemination of research results is a key component of the research continuum and is commonly achieved through publication in peer-reviewed academic journals. However, issues of poor quality reporting in the research literature are well documented. A lack of formal training in journalology (i.e., publication science) may contribute to this problem. To help address this gap in training, the Enhancing the QUAlity and Transparency Of health Research (EQUATOR) Canada Publication School was developed and facilitated by internationally-renowned faculty to train researchers and clinicians in reporting and publication best practices. This article describes the structure of the inaugural course and provides an overview of attendee evaluations and perspectives.

**Key highlights:**

Attendees perceived the content of this two-day intensive course as highly informative. They noted that the course helped them learn skills that were relevant to academic publishing (e.g., using reporting guidelines in all phases of the research process; using scholarly metrics beyond the journal impact factor; open-access publication models; and engaging patients in the research process). The course provided an opportunity for researchers to share their challenges faced during the publication process and to learn skills for improving reproducibility, completeness, transparency, and dissemination of research results. There was some suggestion that this type of course should be offered and integrated into formal training and course curricula.

**Implications:**

In light of the importance of academic publishing in the scientific process, there is a need to train and prepare researchers with skills in Journalology. The EQUATOR Canada Publication School provides an example of a successful program that addressed the needs of researchers across career trajectories and provided them with resources to be successful in the publication process. This approach can be used, modified, and/or adapted by curriculum developers interested in designing similar programs, and could be incorporated into academic and clinical research training programs.

## Background

Dissemination of study results is a key component of the research-to-practice continuum [1]. One traditional and common method of disseminating research findings to the scientific community is through publications in peer-reviewed academic journals. However, only half of completed research projects are eventually published [2–5], and of those that do manage to be published, many are fraught with errors and/or are poorly reported [6]. Together, these issues of publication bias and poor quality reporting contribute to a lack of transparency and prevents reproducibility and critical appraisal of study results, rendering them unusable [7]. Common reporting deficiencies include the provision of incorrect or misleading information (e.g., incorrect statistical analyses), missing or incomplete information (e.g., missing treatment or outcome details in methods), inconsistent information (e.g., between trial protocols and reports), and poorly written or inappropriately used tables, text, or figures [6]. Collectively, these problems highlight numerous avoidable challenges in the scholarly publication of research.

Currently, little is known about how to best train authors for academic publishing [8] and authors instead typically develop these skills through a process of trial and error [9] and/or from their academic supervisors [10]. Globally, national granting agencies are increasingly specifying that grantees disseminate their research results to the widest possible audience at the earliest opportunity to improve the sharing and uptake of research results (e.g., [11]). To ensure their research findings are effectively disseminated, as well as compliance to these types of granting policy mandates, it is essential that researchers are educated on journalology best practices and that they finesse skills related to writing and publication. One freely accessible online resource that is available to support researchers in this respect is the Enhancing the QUAlity and Transparency Of health Research (EQUATOR) Network [12, 13].

The EQUATOR Network is an international collaboration that promotes the transparent and accurate reporting of research and provides a searchable website that includes a library for research reporting guidelines, as well as practical help and resources (e.g., toolkits) for writing and peer-reviewing research articles. Reporting guidelines are tools that authors can use as they draft their manuscripts to ensure that all relevant information pertaining to their research is articulated in a standardized manner [12]. The EQUATOR Network’s reporting guidelines facilitate the critical appraisal of published studies and increases the reproducibility, completeness, and transparency of research reports [12, 14]. To further facilitate improvements in reporting quality, journal publishers are increasingly requiring that these checklists accompany manuscripts submitted by authors [12, 14, 15]. The Network includes centres in Australasia [16], Canada [17], France [18] and the United Kingdom [19]. It also hosts courses and workshops internationally to help researchers improve their research reporting and dissemination skills such as the five-day UK EQUATOR Center Publication School [20]. In 2017, researchers at The Hospital for Sick Children in collaboration with the EQUATOR Canada Centre organized and led the inaugural EQUATOR Canada Publication School.

The purpose of this manuscript is to: 1) to describe the inaugural EQUATOR Canada Publication School - facilitated by a faculty (collectively referred to as the ‘EQUATOR Canada Publication School Faculty’); and 2) to describe attendee evaluations and perspectives from participating in the EQUATOR Canada Publication School (also referred to as the ‘Publication School’). The authors of this paper include Publication School attendees (JG, AC, SG, AJ, AM) and members of the Publication School Faculty and planning committee (ACT, KC, NB). The views expressed herein represent those of the authors who attended the Publication School as trainees (JG, AC, SG, AJ, AM). Publication School Faculty co-authors (ACT, KC, NB) contributed course content information and aggregate results of all attendee evaluations. In the following sections, we describe the Publication School curriculum and structure; present the main themes of attendee evaluation and feedback; and discuss attendee recommendations proposed at the close of the course.

### About the EQUATOR Canada publication school

The inaugural EQUATOR Canada Publication School took place from October 5–6, 2017 at the Hospital for Sick Children’s Peter Gilgan Centre for Research and Learning in Toronto, Ontario, Canada. The Publication School was funded by the Ontario Strategy for Patient-Oriented Research Support Unit (OSSU, [21]), which is an initiative supported by the Government of Ontario and Canadian Institutes of Health Research that fosters engagement from clinicians, patients, researchers, policy makers, and industry representatives to collaborate and implement Canada’s Strategy for Patient-Oriented Research within Ontario [21]. The EQUATOR Canada Publication School was accredited as a Continuing Professional Development (CPD) course through the University of Toronto for 11-credit hours with the Royal College of Physicians and Surgeons of Canada (Section 1) and the College of Family Physicians of Canada (Mainpro+). Such accreditation is useful for attendees with professional designations that require continuing education credits to maintain their certification and encourages participation by clinician-researchers.

The Publication School was led by expert faculty in publication science and consisted of clinicians, scientists, professors, and communications specialists from major hospitals and academic institutions in Ontario (Table 1). The course content included best-practices to prepare, submit, and peer-review scientific manuscripts, as well as strategies for engaging patients, caregivers, and families in all stages of research development and publication.

**Table 1:** EQUATOR Canada 2017 Publication School Faculty and Planning Committee

Name	EQUATOR Canada Publication School Role and Professional Affiliation(s)
Nancy Butcher, PhD	Co-director, Course faculty, and Planning Committee member
	Senior Research Associate, The Hospital for Sick Children
Martin Offringa, MD, PhD	Co-director, Course faculty, and Planning Committee member
	Senior Scientist & Staff Neonatologist, The Hospital for Sick Children
	Professor, University of Toronto
Kelly Cobey, PhD	Course faculty and Planning Committee member
	Investigator, Centre for Journalology, The Ottawa Hospital
	Adjunct Professor, University of Ottawa
	Staff, EQUATOR Canada Centre
David Moher, PhD	Course faculty and Planning Committee member
	Senior Scientist, Centre for Journalology, The Ottawa Hospital
	Associate Professor and University Research Chair, University of Ottawa
	Director, EQUATOR Canada Centre
Peter Gill, MD, PhD	Course faculty and Planning Committee member
	Pediatric Resident, The Hospital for Sick Children
Richard Glazier, MD, MPH	Course faculty and Planning Committee member
	Family Physician, St. Michael’s Hospital
	Scientist, Li Ka Shing Knowledge Institute
	Professor, University of Toronto
Andrea Tricco, PhD	Course faculty and Planning Committee member
	Scientist, Li Ka Shing Knowledge Institute
	Associate Professor, University of Toronto
Andrea Chiaramida, BA	Course coordinator
	Administrative Assistant, The Hospital for Sick Children
Natasha Saunders, MD, MSc	Course faculty
	Associate Scientist & Staff Physician, The Hospital for Sick Children
	Assistant Professor, University of Toronto
Matet Nebres, BSc	Course faculty
	Senior Manager, Media Relations, The Hospital for Sick Children

Publication School attendees learned about diverse publication science topics through a variety of media (for agenda please see Table 2). Interactive lectures provided guidance about a range of topics related to publication best practices across the research continuum, such as the use of reporting guidelines in academic writing, publication ethics, choosing an appropriate academic journal for submission, how to identify and avoid predatory journals, scholarly metrics, and how to give and respond to peer-review. Attendees were also provided with opportunities to engage in active learning and group activities to facilitate their learning (Table 3). For instance, Dr. Cobey complemented her ‘Tools for Transparency’ lecture by having attendees visit the Open Science Framework (OSF, [22]) and Open Researcher and Contributor ID (ORCID, [23]) websites to familiarize attendees with these resources and to create user accounts (see Tables 2 and 3). These resources are essential tools that researchers can use to enhance the transparency of their research processes and to claim their unique author identity, respectively. For many attendees, these activities facilitated attendees’ first steps in these areas.

**Table 2:** EQUATOR Canada 2017 Publication School Agenda

Session Number (Time)	Session Title and Teaching Format	Summary of Session Objectives	Facilitator(s)
Day 1			
1 (0900–0915)	Introduction to the course	Introduce the course aims, content, and method of delivery	Nancy Butcher and Martin Offringa
2 (0915–0945)	Reporting guidelines and their implementation *Interactive lecture*	Introduce reporting guidelines and where to find them, and discuss the difference between endorsement and implementation of reporting guidelines	David Moher
3 (0945–1100)	Writing an article that is fit for purpose using reporting guidelines *Interactive lecture and small group discussion*	Define what is meant by “fit for purpose”, introduce resources for writing an article that is fit for purpose, and outline how to incorporate fit for purpose writing into manuscript writing	Kelly Cobey
4 (1115–1200)	Tools for transparency *Interactive lecture and workshop*	Discuss the importance of transparent reporting, identify tools to ensure transparency, and strategies for integrating transparency best practices into manuscript writing	Kelly Cobey
5 (1200–1230)	Scholarly metrics *Interactive lecture*	Define publication metrics, identify various types of publication metrics, discuss strengths and weaknesses of using publication metrics	Andrea Tricco and Kelly Cobey
6 (1330–1445)	Selective reporting: Detection, consequences, and solutions *Hands-on exercise*	Identify the types and levels of selective reporting and their consequences, identify tools to prevent, identify, or manage selective reporting	Peter Gill
7 (1500–1530)	Publication ethics, authorship, and research integrity *Interactive lecture*	Pinpoint and describe issues pertaining to publication ethics and integrity, identify methods to prevent and manage issues relating to publication ethics and integrity, discuss how to integrate publication ethics into the workplace	Martin Offringa and Richard Glazier
8 1530–1615)	Science and the media: Cases and resources *Case-based learning*	Highlight strategies of sharing and promoting one’s own research, discuss the impact of social media on healthcare and research, with personal and published examples	Nancy Butcher, Peter Gill, Natasha Saunders, and Matet Nebres
9 (1615–1630)	Closing Q&A and completion of Day 1 course evaluation form	To allow attendees opportunity to seek clarification about any course content and time to complete course evaluations	Nancy Butcher and Martin Offringa
10 (1645–1800)	Patient engagement roundtable and networking event	Attendees applied their learning to three faculty-facilitated scenarios: (1) Why engage patients and barriers to engagement; (2) When to engage patients and how to report in manuscripts/reports; and (3) How to identify patients and engage patients	(1) Richard Glazier and Kelly Cobey (2) Martin Offringa and David Moher (3) Nancy Butcher and Andrea Tricco
Day 2			
- (0900–0915)	Re-cap of Day 1 and Questions	To allow attendees opportunity to seek clarification about Day 1 course content	Martin Offringa
11 (0915–1000)	How do biomedical journals operate? *Interactive lecture*	Explain how biomedical journals operate, identify key actors and their roles, describe the process from submission of a manuscript to publication	Andrea Tricco
12 (1000–1030)	How to choose a journal *Interactive lecture*	Identify tools for journal selection and relevant choices when selecting a journal	Martin Offringa and David Moher
13 (1045–1115)	Peer review: Roles of peer reviewers *Interactive lecture*	Describe types of peer review, distinguish between useful and non-useful peer review, identify methods of performing high quality peer review, discuss how to incorporate reporting guidelines into the peer review process, discuss criticisms of the peer review process	Martin Offringa and Richard Glazier
14 (1115–1200)	What makes a good peer review? *Panel discussion (audience Q&A)*	Bring awareness to the characteristics of good peer review, identify unhelpful peer review techniques, discuss where to find reporting guidelines and how to use for a peer review	David Moher, Martin Offringa, Andrea Tricco, Peter Gill, and Richard Glazier
15 (1300–1445)	Fees, predatory journals, open access, and traditional publication models *Case based learning*	Familiarization with the different types of publication models, introduce methods of complying with open access publishing requirements, understand the concept of predatory journals and how they can be identified	Kelly Cobey
16 (1500–1545)	Engaging patients and parents: From design to publication *Interactive lecture with attendee presentations and group discussion*	Explain the concept and importance of patient-oriented research, discuss when to perform patient oriented research, describe how to identify and engage patient stakeholders. Attendees summarized and presented the key messages learned during Session 10.	Andrea Tricco
17 (1545–1600)	Wrap up and completion of Day 2 course evaluation form	To allow faculty and attendees to add any final thoughts or questions about the course content	All

**Table 3:** Summary of interactive learning activities at the EQUATOR Canada 2017 Publication School

Session	Title	Activity description
3	Writing an article that is fit for purpose using a reporting guideline	Peer review and assessment of fit for purpose writing of a published cohort study through application of the STROBE reporting guideline
4	Tools for Transparency	Familiarization with Open Science Framework and ORCID to promote transparency in research methodology
6	Selective reporting: Detection, consequences and solutions	Detection of selective reporting by comparing reported outcomes in a published study protocol versus the published clinical trial report
10	Patient engagement roundtable and networking event	Attendees rotated between three tables led by two faculty each and brain-stormed ideas and thoughts on (1) Why engage patients and barriers to engagement; (2) When to engage patients and how to report in manuscripts/reports; and (3) How to identify patients and engage patients. Attendees voted at each table on the top five take-home messages and nominated a table “reporter” to present their results to the course attendees during Session 16.
15	Fees, predatory journals, open access, and traditional publication models	In groups, attendees applied their understanding of predatory journals in an examination of three faculty-selected journal articles
16	Engaging patients and parents: From design to publication	Group discussion on engaging patients and parents, with student presentation of take-home lessons learned from Session 10

Abbreviations: *STROBE* = Strengthening the Reporting of Observational Studies in Epidemiology, *ORCID* = Open Researcher and Contributor ID

The Publication School attendees were also provided with a number of networking opportunities. Short networking opportunities occurred between lectures and interactive sessions (e.g., during nutrition breaks and lunch) as attendees mingled with each other and Publication School Faculty throughout the day. The wrap-up event for Day 1 was a patient engagement roundtable and networking event. During this event, attendees applied their learning to three faculty-inspired scenarios: Why engage patients and barriers to engagement; When to engage and how to report in manuscripts/reports; and How to identify patients and engage patients. Each scenario was presented by two faculty members who facilitated discussions with groups of attendees at separate tables in a communal area of the venue (see Tables 2 and 3 for details). These discussions were timed so as to allow attendee groups an equal amount of time at each table. At the end of each interval, attendees ranked their top five messages and individual attendees volunteered to collate these ranked responses and report to the entire class during Session 16. Following this engagement roundtable and networking event, attendees and faculty went to a local restaurant for dinner, where they continued to network in an informal and sociable manner.

Attendees and faculty used the hashtag #EQPubSchool to communicate about the course [24]. A few examples include: “#EQPubSchool The Plenary on how to engage patients in the publishing process is coming up shortly. @EQUATORNetwork @dmoher @OSSUtweets”; “Spent the past two days attending #EQPubSchool in Toronto. Excited to apply the tools and tips to all things research. First step #reportingguidelines @EQUATORNetwork”. When the EQUATOR Canada Publication School came to a close, attendees were provided with an opportunity to stay in contact with each other and Publication School Faculty by joining a private LinkedIn group. This provided everyone with a networking opportunity to maintain long-term connections with current course participants, and to create a larger network with attendees of future EQUATOR Canada Publication School participants.

### EQUATOR Canada publication school application process and course attendees

Information about the launch of the EQUATOR Canada Publication School was disseminated through various channels. E-mails were sent to universities across Canada and their respective medical schools and departments. Publication School information and the accompanying application form were posted on Cvent (www.cvent.com), an online event registration and management system. Social media platforms including Twitter and LinkedIn were also used to publicize the Publication School and application process.

Of the 65 individuals who applied to the inaugural Publication School, 34 were competitively selected using a comprehensive application process. This process required attendees to indicate: their primary role (e.g., clinician, graduate student, etc.); their research interests and current studies; whether they engage patients and families in their research; their use of social media for research dissemination; their purpose for attending the Publication School; their level of familiarity with publication science (e.g., reporting guideline usage, publication metrics); and topics or skills they would like to learn about or acquire from attending the Publication School. Applications were evaluated on a continuing basis and were reviewed by two to three planning committee members (e.g., the course co-directors and course co-ordinator). A consensus decision on acceptance was reached for all applicants.

The majority (24/34, 70.6%) registered as trainees (defined as graduate students, postdoctoral or clinical fellows, and medical students). The other registrants included physicians (*n* = 5), research associates (*n* = 2), and a scientist, a lecturer, and a research administrator. Two registrants did not attend the course. Of the 32 course attendees, 24 were female (75%). Attendees were students or affiliated with medical and academic institutions that included The Hospital for Sick Children (*n* = 9), University of Toronto (*n* = 5), The Ottawa Hospital (*n* = 3), McMaster University (*n* = 3), Bruyére Research Institute (*n* = 2), Cancer Care Ontario (*n* = 2), Children’s Hospital of Eastern Ontario (*n* = 2), Queen’s University (*n* = 2), the Centre for Addiction and Mental Health (*n* = 1), McGill University (*n* = 1), Université Laval (*n* = 1), and World Child Cancer (*n* = 1).

Five trainees (*n* = 3 female) were awarded competitive scholarships to help offset the costs of attending the Publication School. The recipients were from McMaster University (*n* = 2), Queen’s University (*n* = 1), Université Laval (*n* = 1) and The Ottawa Hospital (*n* = 1).

### The EQUATOR Canada publication school attendee evaluations and perspectives

At the end of the Publication School, attendees provided verbal feedback during the course wrap-up (see Table 2). Nearly all (28/32, 87.5%) completed anonymous written course evaluations that were collected by Publication School Faculty. Of those that completed the course evaluation, most (17/28, 60.7%) indicated less than 5 years of publication experience; four (14.3%) reported none. There were seven attendees with five to 10 years of publication experience. The completion rate of each item on the written evaluations ranged from 66 to 88% and overall the evaluations indicated that attendees perceived the Publication School content as well-organized, and highly relevant, and valuable to their diverse fields of research and clinical practice. All sessions and faculty were rated on average as between “very good” and “outstanding” on a five-point Likert scale. Of the attendees who provided general comments, many (11/19, 57.8%) indicated an appreciation for the expert mentorship of the Publication School Faculty and described them as e.g., “engaging”, “enthusiastic”, “knowledgeable”, and “responsive”.

Notably, verbal feedback included the suggestion by several participants that this type of course should be offered and integrated into formal training and course curricula. One participant commented in the written evaluation that “Would have liked to have done this course during residency training…we really don’t get much/any formal training in this area. … Given that the field is evolving/not static, course will be relevant for a long time to come.”

Overall, Publication School attendees gained knowledge and learned a diversity of skills relevant to academic publishing (e.g., strategies and resources to maintain integrity of the scientific record; issues relevant to publication ethics, integrity and bias; selecting appropriate publication models; and how to implement the journalology process from manuscript submission to publication). The learning topics that resonated most among these attendee authors are summarized in the following sections and are complemented by the data provided within course evaluations. We believe these perspectives and lessons most poignantly describe our learning from the EQUATOR Canada Publication School and will provide readers with a summary of the valuable insights gleaned about academic publishing.

#### Using reporting guidelines in all phases of the research process

The results of the written course evaluations (*n* = 28) revealed that attendees agreed or strongly agreed that the course enhanced their knowledge and that content was relevant to their research practices. The majority (26/28, 92.8%) of attendees indicated that they would make changes to their research publication practices. Attendees were asked to list changes that they would make in their research practices (e.g., manuscript design and publication) as a result of participating in the course, and notably, the majority (19/28, 67.9%) self-identified the use reporting guidelines as an area of practice change. This is an important change for researchers to adopt, especially those beginning in their career, as reporting guidelines provide a standardized structure for the publication of research methods and results [12] that are increasingly being endorsed and required by academic journals during the manuscript submission and peer-review process in order to improve the quality of research reporting [12, 15]. The attendees acknowledged the availability and diversity of reporting guidelines offered through the EQUATOR Network [13] and the relevance of these guidelines to all phases of the research process. For example, using reporting guidelines to draft a study protocol ensures that all relevant content is included in the protocol, resulting in a more comprehensive study design. Furthermore, publishing such as a protocol prior to carrying out the study facilitates readers’ identification of selective reporting and critical appraisal of study results. Participants noted, for example, that they would “use reporting guidelines when designing my studies”, “always use ‘Reporting Guidelines’ while writing and reviewing papers”, and “use reporting guidelines for every paper (not just RCT)”.

Attendee authors value the use of reporting guidelines to facilitate peer-review of manuscripts submitted for publication. This was an important topic for this group of attendees, the majority of which were trainees and early in their career of academic publishing. We, the next generation of peer reviewers, appreciated the interactive learning session during Session 3 (see Tables 2 and 3) that provided us with some experiential insight into the world of peer-review. In Session 3’s activity, we used an applicable reporting guideline (STROBE [Strengthening the Reporting of Observational Studies in Epidemiology; [25]) to critically appraise a published article for its alignment with the STROBE elements. The activity clearly illustrated for us the variation of reporting clarity that can exist in a single publication and therefore the importance of using reporting guidelines to guide the peer-review process. In the written course feedback, some (3/19, 15.7%) attendees suggested that further information about how to improve peer-review skills be considered for inclusion into future Publication Schools, however, one attendee commented that “the time spent on peer-review was longer than necessary”. For attendees wishing to learn more about the peer-review process and/or improve their peer-review skills, they were encouraged to access reputable online resources such as those made available by the Publons community [26].

#### Using metrics beyond the impact factor

Another common area of self-identified research practice change in the course evaluations was in the area of journal selection. Nearly half (11/26, 42.3%) of the respondents who identified what they would do different in their research practice wrote that they would make changes to their process of selecting a journal to submit a manuscript. A new revelation for attendees was consideration of scholarly metrics beyond the popular journal impact factor (JIF). Attendees were introduced to the limitations of JIFs, for example, that 65–75% of articles in any given journal have a citation count below the journal’s impact factor [27]. Indeed, a declaration advocating for “robust and time-efficient ways of evaluating research” whereby researchers do not rely on JIFs, was initiated in 2012 [28] and was introduced to attendees during this session. This declaration, referred to as the Declaration On Research Assessment (DORA, [28]) is endorsed by international groups and academics who recognize the deficiencies of the journal impact factor and support the need for improved evaluation of research output. Learning about scholarly metrics apart from the JIF was a source of important learning for attendees, who cited an appreciation for and intended use of other metrics for journals (SNIP [Source Normalized Impact per Paper]), articles (e.g., Altmetric) and researchers (e.g., H-Index).

#### Open-access and alternative avenues for publication

Another important source of learning for attendees was about open-access publication models. Attendees were introduced to the availability and procedures for open-access publishing and learned about information and resources useful to navigate this process. We appreciated learning about resources such as the Directory of Open Access Journals (DOAJ, [29]), which is a useful resource against predatory journals. DOAJ is a freely accessible online directory of reputable, open-access, peer-reviewed journals. Learning about these resources and other relevant information (e.g., article processing fees [APCs], archiving, repositories) were invaluable to facilitate the processes that we will follow when preparing and submitting our manuscripts for publication.

Attendees also appreciated learning about research dissemination methods beyond traditional academic publishing, as reflected in the course evaluation forms. The majority of respondents (20/28, 71.4%) reported that they are more likely to use social media to disseminate results of their research after completing the Publication School. Our discussion about useful media options to disseminate research included: Twitter and other social media platforms; blogging; podcasts; writing for institutional or association newsletters or magazines; and opportunities for dissemination through participation (e.g., public disease ‘awareness weeks’, at local library activities, Café Scientifiques). We found it particularly useful to hear from a local researcher and member of the Publication School Faculty, Dr. Natasha Saunders, who had her research findings recently highlighted on television and other media. Hearing Dr. Saunders’ perspectives about contacting and preparing for this media coverage broadened our awareness for using dissemination strategies to reach beyond academic audiences. For those of us who are new adopters of social media dissemination strategies, we learned about useful resources to facilitate our understanding about how media can be used (e.g., TED Talks or lynda.com about how to use Twitter).

## Discussion

In this paper, we have described the inaugural EQUATOR Canada Publication School, and have included the perspectives of Publication School attendees. During this EQUATOR Canada Publication School, attendees were introduced to the principles of publication science and exposed to a wide range of topics, practices, and resources related to the publication process. The Publication School provided a platform that fostered constructive dialogue about contemporary challenges facing the broader scholar community (e.g., oligopoly of commercial publishers in the biosciences, democratization of knowledge, open-access, and green archiving); addressed early-career researchers’ concerns and learning needs across disciplines; and equipped attendees with resources to facilitate success with the publication process. In this way, we – attendees of the inaugural EQUATOR Canada Publication School - gained an understanding about the myriad of factors that can influence and impact integrity of the scientific record, and how to access strategies and resources to manage these factors.

Evaluations submitted by attendees provided evidence that the content and structure of the Publication School was well-received and highly applicable to their respective fields. The majority of attendees reported that the course influenced them to make changes to their current research practice, whether that be using reporting guidelines, using the media to disseminate research, or generally being more transparent with their research processes. Most attendees provided perspectives about the impact of the Publication School that resonate with existing recommendations from the EQUATOR Network and experts in the field of publication science. These perspectives included the adoption and use of reporting guidelines in all phases of the research process, using metrics beyond the journal impact factor, and using open-access and alternative avenues for dissemination. Feedback received from Publication School attendees demonstrate the usefulness of this approach and relevance for researchers across their career trajectories (e.g., from trainees to clinician-scientists or academics).

### Limitations and recommendations

Although all available (*n* = 28) attendee feedback was reviewed while preparing this manuscript, the perspectives reflected also reflect those of the attendee authors (JG, AC, SG, AJ, AM). It is recognized that the perspectives presented herein may not reflect all Publication School attendees who did not co-author this manuscript.

The evaluative data from attendees presented in this paper were only collected at end of the Publication School. In this way, pre- and post- comparisons of Publication School learning were not possible, and any changes in research publication, review, or dissemination practices occurring since the Publication School were not evaluated and are outside the scope of this paper. A limitation of the course identified in attendees’ evaluations was that the Publication School curriculum largely focused on the biomedical sciences and was most applicable to quantitative research paradigms. Colleagues belonging to Nursing, Rehabilitation Science, Sociology and other similar disciplines suggested that the curriculum could be broadened to include topics such as research integrity and bias in qualitative or mixed-method research.

Notably, some early-career attendees recommended that the Publication School curriculum could be offered as two separate programs in the future. For example, a novice program could be offered to attendees with little to no publication experience, whereas an advanced level could be offered to attendees with publication experience and needing exposure to advanced topics. This phased approach may provide a more suitable curriculum to meet the needs and experiences of prospective attendees. Other attendees felt that the Publication School duration could have been longer (i.e., more than 2 days to cover the course curriculum).

A notable recommendation proposed by one attendee was that organizers of the Publication School reach out to program directors at universities to facilitate the incorporation of Publication School content within curricula to improve the journalology skills among novice researchers.

## Conclusion

Publication of manuscripts in a scholarly journal is a commonly used method of disseminating research findings that is not without limitations and bias. The EQUATOR Canada Publication School represents one approach to help improve the transparency and quality of research reporting through education and outreach to researchers. Through this course, the EQUATOR Canada Publication School faculty addressed an array of key publication science topics (e.g., use of reporting guidelines, education around predatory journals, and patient engagement in research) through the use of didactic lectures, interactive workshops, and networking activities. This approach could be used, modified, and adapted to reflect the needs of different disciplines and undergraduate and graduate levels. The attendees’ responses to the Publication School indicate that this content fulfills an important unmet need in publication science training among academics. The adoption of formal publication science training promises to help improve research reporting and dissemination skills among researchers in all disciplines of academia.

## Abbreviations

APC: Article processing fees
CPD: Continuing professional development
DOAJ: Directory of Open Access Journals
DORA: Declaration On Research Assessment
EQUATOR: Enhancing the QUAlity and Transparency Of health Research
ORCID: Open researcher and contributor ID
OSF: Open science framework
OSSU: Ontario strategy for patient-oriented research support unit
SNIP: Source normalized impact per paper
STROBE: Strengthening the reporting of observational studies in epidemiology
